# *wnt11f2* Zebrafish, an Animal Model for Development and New Insights in Bone Formation

**DOI:** 10.1089/zeb.2022.0042

**Published:** 2023-02-14

**Authors:** Caroline Caetano da Silva, Agnes Ostertag, Ratish Raman, Marc Muller, Martine Cohen-Solal, Corinne Collet

**Affiliations:** ^1^ INSERM U1132 and Université Paris-Cité, Reference Centre for Rare Bone Diseases, Hospital Lariboisière, Paris, France.; ^2^ Laboratory for Organogenesis and Regeneration (LOR), GIGA-Research, Liège University, Liège, Belgium.; ^3^ UF de Génétique Moléculaire, Hôpital Robert Debré, APHP, Paris, France.

**Keywords:** *wnt11f2*, zebrafish, high bone mass, Wnt pathways, development

## Abstract

Wnt signaling is a key regulator of osteoblast differentiation and mineralization in humans and animals, mediated by the canonical Wnt/β-catenin and noncanonical signaling pathways. Both pathways are crucial in regulating osteoblastogenesis and bone formation. The zebrafish silberblick (*slb*) carries a mutation in *wnt11f2*, a gene that contributes to embryonic morphogenesis; however, its role in bone morphology is unknown. *wnt11f2* was originally known as *wnt11*; it was recently reclassified to avoid confusion in comparative genetics and disease modeling. The goal of this review is to summarize the characterization of the *wnt11f2* zebrafish mutant and to deliver some new insights concerning its role in skeletal development. In addition to the previously described defects in early development in this mutant as well as craniofacial dysmorphia, we show an increase in tissue mineral density in the heterozygous mutant that points to a possible role of *wnt11f2* in high bone mass phenotypes.

## Introduction

Because of the high degree of conservation among species of the Wnt pathway and its extensive homology with its human homolog, zebrafish mutants represent key animal models to unravel the function of the Wnt signaling pathway in development and bone formation.^1^ Both the Wnt pathways play major roles in bone development and bone remodeling. The extracellular Wnt signal stimulates several intracellular signal transduction cascades, including the canonical or Wnt/β-catenin-dependent pathway and the noncanonical or β-catenin-independent pathway, which can be divided into the Planar Cell Polarity (PCP) pathway and the Wnt/Ca^2+^ pathway.^2^

The *wnt11f2* mutant, presents a mutation in a gene related to the Wnt noncanonical pathway. This fish is also known as silberblick (*slb*), and it was first characterized in 1996 in the frame of a large-scale screen for mutants affecting forebrain development by using N-ethyl-N-nitrosourea (ENU) as a potent mutagen to introduce random point mutations in the genome.^3^ Homozygous *wnt11f2* mutant embryos were smaller at 24 hours post fertilization (hpf) than controls and the elongation of the body axis was delayed from the tailbud stage until the early somite stages (10–14 hpf), as indicated by a shorter and broadened notochord and an abnormally shaped prechordal plate.

Further characterization revealed that anterior migration of central nervous system cells was impaired in *wnt11f2* mutants,^4^ thus leading to incomplete separation of the optic stalk^3^ and fusion of the eyes to various degrees depending on the penetrance of the phenotype. For that reason, this mutant was called silberblick (*slb*), which in German means strabismus. At 5 dpf, the jaw was deformed. The mutants displayed a recessive phenotype with variability.

In the year 2000, the locus that affected the *wnt11f2* mutant, then known as *slb*, was found to encode a homolog of the human WNT11 protein, and was thus initially named *wnt11*.^5^ Two mutants, carrying the variants *tx226* (c.669G>A p.Trp 223) and *tz216* (c.463G>T p.Gly 155) in this gene, were analyzed. In addition, the authors showed that microinjecting *wnt11* mRNA into *slb* mutants was able to rescue their phenotype, confirming that the detected mutations in *wnt11* are indeed responsible for the phenotype and suggesting that the *wnt11f2^tx226^* allele represents a loss-of-function mutation rather than a dominant-negative form. Interestingly, microinjection of *wnt11* mRNA into wild-type embryos often resulted in misshapen eyes, indicating that the amount of Wnt11 proteins needs to be well controlled for normal development.^6^ Since then, this mutant fish line has been used extensively to understand the function of Wnt signaling in early development of vertebrates.

In 2019, genomic analysis revealed that the two zebrafish *wnt11* homologs had been named incorrectly: the gene previously known as *wnt11r* (*wnt11*-related) is the true ortholog to the human *WNT11*, thus now called *wnt11*, while the gene previously known as *wnt11* is present in birds and other teleosts but not existing in mammals is now called *wnt11f2*.^7^ Following the zebrafish nomenclature guidelines, a gene should be named after its mammalian ortholog, however, such confusion occurred before the full zebrafish genome had become available because phylogenetic analyses at that time revealed that the closest human family member to *slb* was *WNT11*.^7^

In PubMed, a simple search with the word “silberblick” resulted in 21 publications published between 1996 and 2013; searching for “wnt11 and zebrafish” identified 99 publications, although not all of them are related to zebrafish and the *wnt11f2* mutants; and only one article from 2021^8^ was found using the “wnt11f2” search term. In contrast, the carefully manually curated international zebrafish network ZFIN (zfin.org) currently has 155 citations concerning the *wnt11f2* zebrafish gene. This situation clearly reflects the confusion in comparative genetics and disease modeling due to incorrect nomenclature resulting from the presence of duplicate paralogs in zebrafish following the whole genome duplication in teleosts. Consequently, this review specifically aims to clarify the role of the *wnt11f2* gene because the publications are not well aligned in just one platform.

Therefore, we focused on the characterization of *wnt11f2*. In the first part of this review, we describe the morphological and genetic characterization of *wnt11f2* zebrafish. In the second part, we add some new findings concerning bone formation using this fish model.

## *Wnt11f2* During Gastrulation

Vertebrate gastrulation is a complex morphogenetic process that organizes the embryo proper into the three germ layers: endoderm, mesoderm, and ectoderm.^9^ Several coordinated morphogenetic cell movements take place during gastrulation, including convergence and extension (C&E) movements mainly mediated by the noncanonical Wnt signaling pathway, also known as the PCP pathway. The PCP signaling pathway was first identified in *Drosophila*, and its cellular and molecular regulation is conserved from *Drosophila* to mammals.

During the gastrulation process, mesodermal and neuroectodermal cells move toward the dorsal midline and intercalate with one another, which leads to mediolateral narrowing (convergence) and anterior–posterior lengthening (extension) of the developing embryonic axis.^9,10^ The *wnt11f2* is maternally expressed^11–13^ and is one of the immediate early genes activated in mesoderm induction. The *wnt11f2* is expressed in the dorsal region of the germ ring at sphere/dome stage (4–5 hpf). During gastrulation, *wnt11f2* expression extends to the lateral and ventral germ ring while being downregulated in the shield and its axial derivatives. In addition to the germ ring, at the end of gastrulation, *Wnt11f2* is expressed in restricted areas of the anterior paraxial mesoderm and anterior lateral neuroectoderm.^5^ *Wnt11f2* activity is required for cells to undergo correct C&E movements during gastrulation, as the *wnt11f2^tx226^* mutants present an impairment of the Wnt/PCP signaling leading to a diminished body axis elongation.^14^

A gastrulation phenotype is transiently visible in *wnt11f2* mutants between 10 and 12 hpf: C&E movements of both mesendodermal and neuroectodermal cells are reduced, which results in a shortened and broadened body axis that can be used to identify homozygous mutants at the end of gastrulation.^4^ The prechordal plate in the anterior axial mesendoderm appears to be abnormally shaped; the presumptive neural plate in *wnt11f2^tx226^* embryos appears to be broader during gastrulation. The *wnt11f2* is required in the nonaxial mesoderm to mediate cell intercalation along the anteroposterior axis that contributes to the extension of the body axis during late gastrulation.^4^ Shield and cell transplantation experiments performed by Heisenberg et al, where wild-type or mutant shields, or small groups of lineage-labeled nonaxial mesodermal cells were transplanted into *wnt11f2^tx226^* mutant or wild-type embryos, respectively, to assess the movements, and the final location of the transplanted cells revealed that Wnt11f2 activity is required within lateral tissues of the gastrula, where it regulates mediolateral cell intercalations that underlie C&E movements.^4^

The observation that *wnt11f2* embryos are predominantly affected in anterior regions of the gastrula suggests that other genes are involved in regulating C&E movements in more posterior regions. Therefore, in the absence of Wnt11f2, abnormal extension of axial tissue results in cyclopia and other midline defects in the head.^5^

Also, *wnt5b* partially overlaps functions with *wnt11f2* to regulate C&E movements in lateral domains of the gastrula,^15^ confirming that both *Wnt11f2* and *Wnt5b* are required during gastrulation for proper morphogenesis rather than cell fate specification. The *wnt11f2/wnt5b* double mutants show severe C&E cell-movement defects, and *wnt5b* RNA partially rescues the *wnt11f2* mutant phenotype.^15^ At 3-somite stage, *wnt11f2^tz216^* is required for C&E of both the mesoderm and ectoderm in the anterior and posterior region.^8^

Wnt11f2 also controls the orientation and the velocity of the hypoblast cell migration in the germ ring at the onset of gastrulation; *wnt11f2^tx226^* mutants present slower and less direct migratory movements of these hypoblast cells.^16^ The authors observed that there is a misalignment in the *wnt11f2^tx226^* hypoblast cells during the orientation process and the direction of movement, leading to less efficient movements of hypoblast cells toward the animal pole.^16^

Another defect observed in the *wnt11f2* mutants is the mitotic divisions in the dorsal epiblast cells, which exhibit less-pronounced animal–vegetal axis polarity relative to control cells in both the epiblast and perpendicular planes, meaning that there is a disruption in the cell division orientation in the mutants.^17^ In addition, it has been described that Wnt/PCP signaling plays an important role in the migration of the cranial neural crest^18^; together with other genes from the Wnt/PCP signaling such as *Fzd7* and *Disheveled*, *wnt11f2* controls the polarization of the protrusions formed in the neural crest, which allow the cells to migrate in a directed motion.^19,20^

The Wnt/PCP pathway also plays a role in cell adhesion. The *wnt11f2^tx226^* triggers the local accumulation of Fz7 at cell membranes along with the intracellular mediator Dsh and Wnt11f2 itself, modulating local cell contact persistence to coordinate cell movements during gastrulation.^21^ To summarize, gastrulation is the first large-scale morphogenetic process to occur during development. This process is in part regulated by Wnt/PCP pathway, and disruptions in the regulation of this pathway caused by a mutation in *wnt11f2* in zebrafish cause defects in the mediolateral cell intercalation, the migration of hypoblast cells, the cell division orientation, and in cell adhesion.^15–17,21,22^

## Pharyngula Stage of *wnt11f2* Zebrafish

This stage focuses on the primordia of the pharyngeal arches, present at early times but difficult to distinguish individually. In zebrafish, the pharyngula stage starts at around 24 hpf. The embryo is most evidently now a bilaterally organized creature, entering the pharyngula period with a well-developed notochord and a newly completed set of somites that extend to the end of a long postanal tail. The nervous system is hollow and expanded anteriorly.^23^

The expression of *wnt11f2* in the wild-type zebrafish is at the developing somites and otic placodes at 24 hpf and also in the mesoendoderm and in midline structures during zebrafish heart morphogenesis.^24^ At 24 hpf, in about half of the *wnt11f2* mutant embryos, the retinae are not properly separated anteriorly. The forebrain in *wnt11f2* mutant embryos, although patterned normally, is broadened and shortened, possibly the result of defective C&E during gastrulation.^25^ The axonal scaffold appears normal, with the exception of a slight deformation at the anterior–ventral positions owing to the fusion of the eyes.^4^ It is possible that the major cause of the reduced resolution of bilateral eye fates in this mutant is related to the defect of forebrain morphogenesis.^25^ At 26 hpf, *wnt11f2^tz216^* mutants have normal tails.^26^

At 48 hpf, the eyes are slightly turned inward anteriorly, with no further obvious abnormalities detectable. Heisenberg and Nüsslein-Volhard propose that proper midline morphogenesis is essential for lateralization of the eye position.^4^ Although *wnt11f2* mutants show reduced medial–lateral cell intercalations in both anterior and posterior mesendodermal domains, the extension of anterior regions seems most severely affected.^5^

Assessment of the posterior body length at 48 hpf revealed that *wnt5b* but not *wnt11f2*^tz216^ embryos had significantly shorter body axes than control siblings and that *wnt11f2*^tz216^*/wnt5b* double-mutant embryos had the shortest body axes. Similarly, the gut tube was normal in *wnt11f2*^tz216^ embryos but slightly enlarged in *wnt5b* mutant embryos and significantly widened in *wnt11f2*^tz216^*/wnt5b* double-mutant embryos. These data suggest that at 48 hpf, *wnt5b* but not *wnt11f2* is required for elongation of the body axis and formation of the gut tube, but *wnt11f2* cooperates with *wnt5b* to regulate endoderm morphogenesis.^8^

## Larvae Stage of *wnt11f2* Zebrafish (3 to 30 dpf)

Zebrafish larvae show a clear and distinct swimming pattern in response to light and dark conditions following the development of a swim bladder at 4 dpf. Cartilage cells are distinctive in branchial arches in these later larval stages. The primordium of the operculum extends posteriorly to cover the first or even the second branchial arch. The first visible bone in zebrafish, the transversely oriented cleithrum, appears at 3 dpf.

Craniofacial cartilage elements are derived from the cranial neural crest. As mentioned above, in zebrafish, Wnt/PCP signaling plays a role in the migration of the cranial neural crest cells.^18^ As neural crest cells migrate, they extend polarized protrusions allowing the cells to migrate in a directed motion. The Wnt/PCP elements *Wnt11f2*, *Fzd7*, and *Disheveled* control at least in part the polarization of these protrusions.^19,27^ Between 55 hpf and 3 dpf, *wnt11f2* is expressed in the pharyngeal arches.^28^ In addition, *wnt11f2* has a complex expression pattern, including expression in head neural crest that might eventually form the anterior basicranium. However, the expression domain is within early paraxial mesoderm, before mesoderm has migrated to reach where the head will form,^5^ with this observations are possible that genes responsible for severe craniofacial phenotypes might be remote to cartilage development itself.^29^ At 48 hpf the wnt11f2 is expressed in the lower jaw.^30^ By 4 dpf, most of the craniofacial cartilage elements of the zebrafish have formed.^31^ Indeed, the Wnt signaling pathways are known to regulate bone homeostasis.

Wnt11f2 function in zebrafish cartilage formation is to position the initial bilateral sites of chondrogenesis of the anterior basicranium and specify where migrating neural crest cells will settle down at early stages determining the fate of the cartilage morphology.^29^ In *wnt11f2* mutant zebrafish, the jaw is deformed.^3^ To understand what is causing this bone and cartilage defect, Sisson et al, in 2015,^28^ marked the outline of chondrocytes to assess the gross morphology of the cartilage elements. In *wnt11f2^tz216^* fish at 4 dpf, the cartilage elements, especially the ceratohyal and Meckel's cartilage, were greatly deformed. The mutants showed disrupted placement of many of the cartilage elements derived from the premandibular, mandibular, and hyoid arches. However, the stacking of the chondrocytes seemed normal. In the *wnt11f2* mutants, the prechordal plate is severely defective, which impairs the extension of axial tissues leading to defects in the head cartilage formation, chondrogenesis occurring near the end of embryogenesis.^29^

The *wnt11f2* mutants deform a bilateral organization in the mutants into a one-dimensional array just along the midline. Wnt11f2 is important to determine the position of the initial normally bilateral sites of chondrogenesis of the anterior basicranium.^29^ Furthermore, molecular studies reveal that *wnt11f2* function in Wnt noncanonical signaling pathway is crucial for embryonic midline development.^24^

Of note, the *wnt11f2^tz216^* eye phenotype can vary interindividually, together with the severity of the craniofacial defects. To determine whether the severity of the eye phenotype was related to the cartilage placement phenotype, Marlow et al compared embryos manifesting full cyclopia (class 5) to those with lesser degrees of synophthalmia (classes 2 and 3).^32^ The authors found a clear correlation between the progression of the eye-fusion phenotype and the disruption of the shape of the cartilage elements, especially the ceratohyal. However, the ability of the chondrocytes to stack was not disturbed. This observation suggests that the craniofacial defect seen in the *wnt11f2^tz216^* mutant fish is due primarily to the eye field separation defect displacing the cartilage elements.

**FIG. 1.**
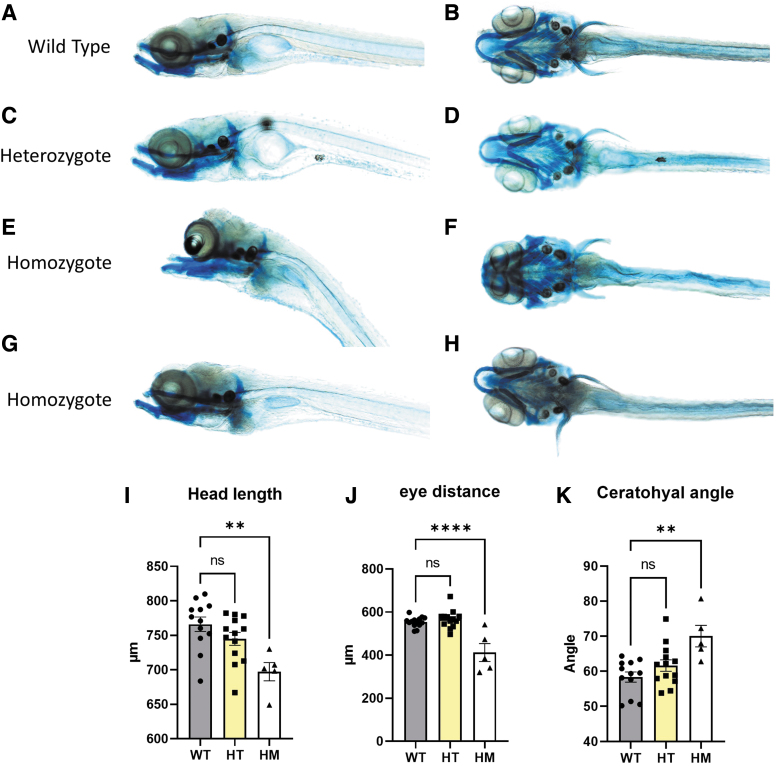
Homozygous *wnt11f2^tx226^* larvae present a strong midline craniofacial dysmorphia. WT **(A, B)**, heterozygous *wnt11f2^tx226^* **(C, D)**, and homozygous *wnt11f2^tx226^* **(E–H)** zebrafish, stained with Alcian *Blue*. Head length **(I)**, distance between the eyes **(J)**, and ceratohyal angle **(K)**. *n* = 14 WT *n* = 13 heterozygous *n* = 5 homozygous. Data are mean ± SEM. **p < 0.01, ****p < 0.0001. SEM, standard error of the mean; WT, wild type.

To obtain more information about the role of *wnt11f2* signaling in zebrafish cartilage formation, we performed Alcian Blue staining, as described,^33^ to check the cartilage formation at 10 dpf in *wnt11f2^tx226^* mutants (a material and methods section is available in the supplementary data). To investigate a possible gene dosage effect, we analyzed in parallel heterozygous and homozygous *wnt11f2^tx226^* mutants. Compared with wild-type (Fig. 1A, B), homozygous mutants showed striking craniofacial malformations (Fig. 1E, F) mainly in the dorsal part, whereas cartilage formation in the lower jaw appeared surprisingly normal. Precise measurements revealed that both the head length and the distance between the eyes were significantly decreased (Fig. 1I, J), and the ceratohyal angle was increased (Fig. 1K) in mutant zebrafish. Meckel's opening and the distance between Meckel's opening and the ceratohyal did not differ in mutant and wild-type fish. In addition, we noted a large variability in the phenotype of individual larvae (Fig. 1G, H), probably indicating a modifier effect due to their individual genetic background.

These results are consistent with the hypothesis that Sisson et al^28^ suggested: *wnt11f2* mutations affect craniofacial development indirectly as a result of synophthalmia leading to improper head morphology, which could explain the normal cartilage formation in the lower jaw. In contrast, wild-type and heterozygous *wnt11f2^tx226^* mutants did not differ in cartilage formation in the lower jaw (Fig. 1A–D), which further supports the conclusion of a recessive, loss-of-function mutation.

The first description of the *wnt11f2* zebrafish model discussed that the eye phenotype seemed to be due to aberrant differentiation of midline tissue restricted to the anterior forebrain.^3^ The expression domain of *shh* in the anterior-most part of the neural keel overlying the prechordal plate was shortened in *wnt11f2* mutants, but at later stages of development (16 hpf), the *wnt11f2* embryos showed normal expression of *shh*. The primary defect in *wnt11f2* may be a reduction in medial–lateral intercalation of cells in the axial mesendoderm. Our results and those of Sisson et al^21^ confirm that *wnt11f2* mutations affect craniofacial development indirectly through cyclopia resulting in incorrect head morphology. There is a link between the degree of severity of cyclopia and the degree of severity of craniofacial cartilage defects.

**FIG. 2.**
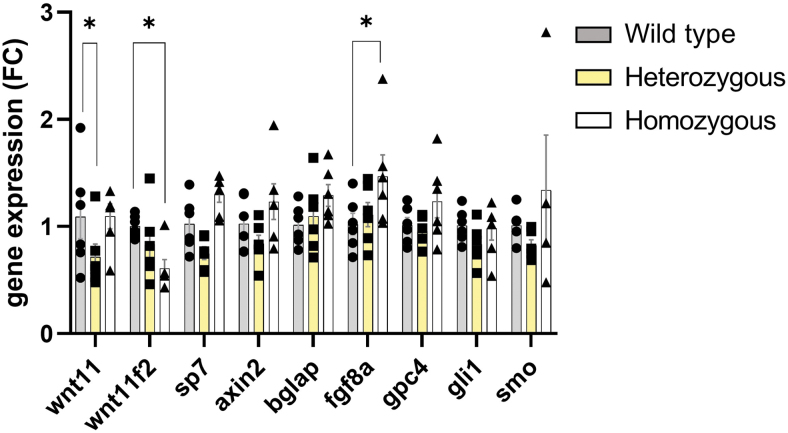
Expression of osteoblast differentiation markers and *shh* target genes in zebrafish. RT-qPCR analysis of mRNA levels of whole head from 6-dpf larvae. Data are mean ± SEM. *p < 0.05. RT-qPCR, reverse transcription-quantitative polymerase chain reaction.

To better understand the craniofacial anomalies in the zebrafish mutant, we tested the expression of some major genes involved in holoprosencephaly. The gene expression analysis comparing wild type, heterozygous, and homozygous revealed a significant increase in *fgf8a* gene expression (Fig. 2). Primers used for zebrafish reverse transcription-quantitative polymerase chain reaction are displayed in Supplementary Table S1. The relation between the *wnt11f2* and FGF pathway is unknown, but frontonasal skeleton and optic capsular development depended on *Fgf8* in mouse and chicken.^34,35^ In addition, both are important in the neural crest development in zebrafish.^36^ We considered a possible compensation in *wnt11* for *wnt11f2* mutants but found no difference at 6 dpf. The compensation process could be related to tissue specificity or be time point relative.

## Adult *wnt11f2^tz216^* Mutant Zebrafish

**FIG. 3.**
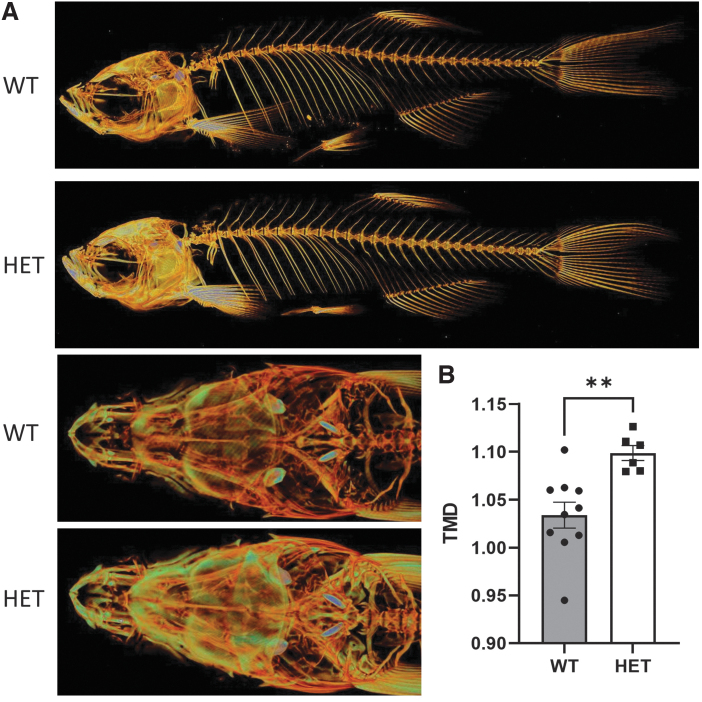
Heterozygous *wnt11f2^tz216^* shows increased bone mass. **(A)** Micro-CT in wild-type and heterozygous adult *wnt11f2^tz216^* siblings in the whole body and skull. **(B)** TMD was analyzed by using CTAN. Data are mean ± SEM. **p < 0.01. CT, computed tomography; HET, heterozygous; TMD, tissue mineral density.

There is no characterization available for *wnt11f2^tx226^* and *wnt11f2^tz216^* zebrafish mutants in adults. Not surprisingly, homozygous mutants with a severe phenotype do not survive to adulthood; however, we were able to perform micro computed tomography analysis of adult *wnt11f2^tz216^* heterozygous zebrafish.^37^ Whole-body scans with a spatial resolution of 7 μm were acquired at 70 kV and 100 μA with a 0.5-mm aluminum filter, revealed a significant increase in bone mineralization over the entire length of the heterozygous *wnt11f2^tz216^* zebrafish, including the head, axial skeleton, and tail fin, and thickening of many individual bone elements (Fig. 3A). We also observed a significant increase in tissue mineral density in the vertebrae in heterozygous zebrafish (Fig. 3B). These individuals presented normal development, morphology, and reproduction. Therefore, these heterozygous mutant fish may represent an excellent model for a high bone mass (HBM) phenotype.

## *Wnt11f2* and the Wnt Pathways

After the discovery that a wnt11f2 truncated protein led to the *slb* zebrafish phenotype, experiments to understand how the pathway works were performed. The *slb* phenotype could be rescued by injection of *wn11f2* mRNA; however, activation of the canonical Wnt pathway by injection of mRNA for the intracellular mediator of the pathway or for an activated form of β-catenin (Δ*N*-β-*catenin*) only dorsalized the embryos. Blocking canonical Wnt signaling by injecting a dominant-negative form of Tcf3 (ΔN-Tcf3) led to ventralization, without rescuing the *wnt11f2* phenotype.^5^ In addition, the authors working with the *wnt11f2* morpholino model showed that when they injected disheveled (dvl) protein, found a cardiac phenotype rescue and when they blocked dvl, they found a phenocopy of the *wnt11f2* phenotype. These observations confirmed that dvl acts downstream of *wnt11f2* in the Wnt noncanonical pathway.^5,24^

Similar observations were made when *ror2* was disrupted in zebrafish, a phenocopy of *wnt11f2* was also found. Moreover, coexpression of low-dose *ror2-TM* mRNA together with injection of *wnt11* morpholino, led to a more severe C&E and eye defects.^38^

The Wnt/PCP signaling pathway is required during the gastrulation process, controlling tissue polarity and cell movement by activating RhoA, c-Jun N-terminal kinase (JNK), and nemo-like kinase (NLK) signaling cascades. The *wnt11f2* is one of the representative noncanonical Wnts transducing PCP signals through fzd7 receptor.^19^

**FIG. 4.**
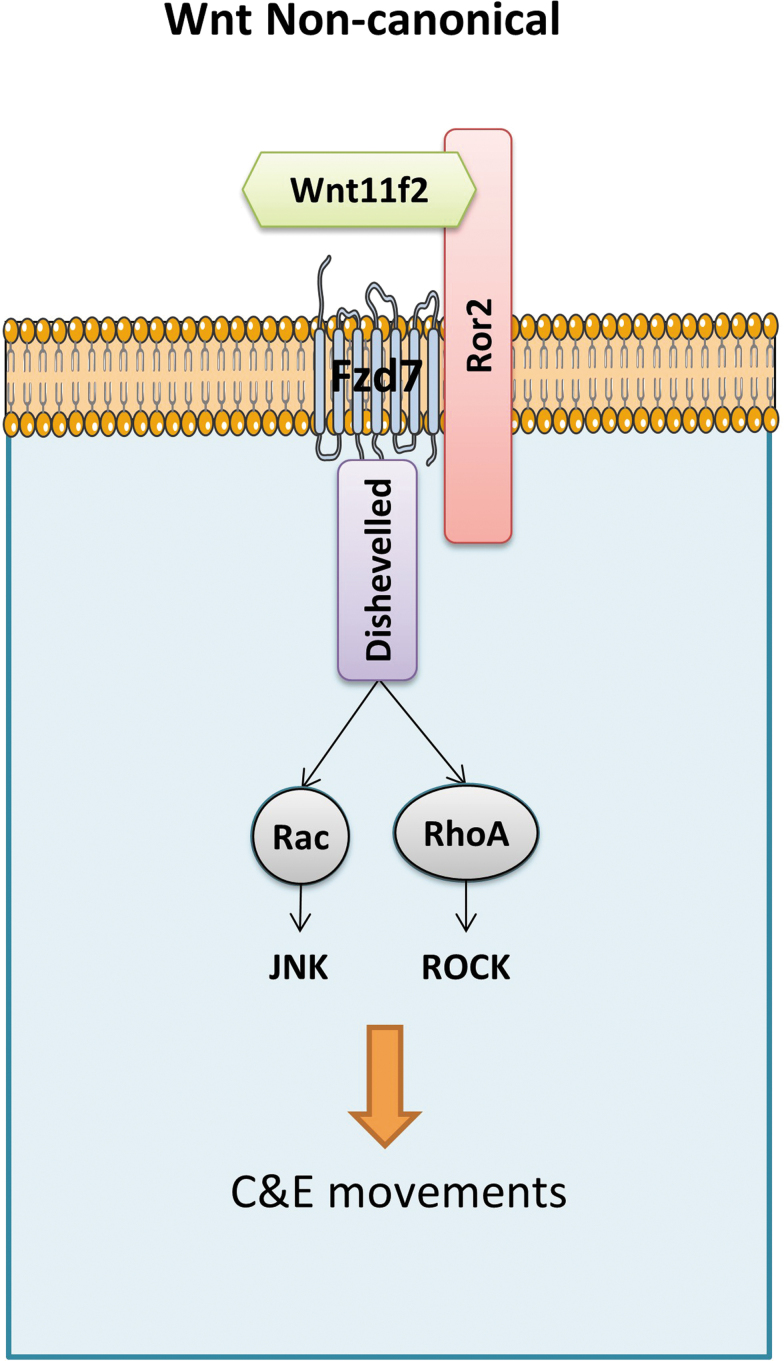
Simplified model of Wnt/PCP noncanonical pathway. Wnt/PCP signaling is induced by noncanonical Wnt ligands, wnt11f2 binding to Fzd7 receptor, and coreceptors such as Ror2. Disheveled-activated downstream effector proteins relay the signal for C&E movements. C&E, convergence and extension; PCP, Planar Cell Polarity.

A summary of the major actors during zebrafish gastrulation can be found in Figure 4.

In bone tissue, the PCP signaling pathway and tissue polarization have been associated with embryonal bone and joint formation, which involves cell migration, elongation, and gradient-dependent differentiation. For example, PCP is crucial during embryonal long bone cartilage elongation along the proximal–distal axis.^39,40^ This gradient, in turn, induces a gradient in the Vangl2 phosphorylation end establishment of tissue polarity.^41^ Similar effects were observed for Wnt5b, with PCP activation involved in Wnt5b-induced cell migration and chondrocyte differentiation.^42^

The *wnt11f2/wnt5b* double-mutant phenotype resembles the phenotype of embryos homozygous for *kny*, which encodes glypican 4 (*gpc4*). Coexpression of *gpc4* RNA potentiated the activity of *Wnt11f2* to rescue the *wnt11f2* C&E phenotype.^43^ Furthermore, *wnt11f2/gpc4* double mutants displayed a more severe phenotype than either mutant alone, including complete cyclopia featuring a single eye, which indicates that *gpc4* acts as a positive regulator of *Wnt11f2*. The *gpc4* is required for C&E of both the mesoderm and endoderm and interacts with *wnt11f2*.^43^ The *gpc4* zebrafish mutants present broader and shorter body axis, the mutation is lethal after 5–7 dpf, but rescue experiments revealed that *gpc4* has a role in regulating cartilage cell polarity, chondrocyte stacking, and endochondral ossification.^44^

The transgenic expression of GFP-*gpc4* in the endoderm of *gpc4* mutants rescued C&E defects in all germ layers. The rescue of mesoderm was likely mediated by *Wnt5b* and *Wnt11f2* and depended on signaling filopodia. *Gpc4* can physically bind both *Wnt5b* and *Wnt11f2* and regulates the formation of the filopodia that transport *Wnt5b* and *Wnt11f2* to neighboring cells.^8^

*Wnt5b* or *Wnt11* initiate the noncanonical Wnt signaling pathway by binding to fzd2 and 7 receptors to regulate C&E movements in zebrafish.^15^ *Wnt11f2* and *wnt11* show spatial and temporal patterns of expression compatible with a role during heart-tube assembly and their transcripts are expressed in neural ectoderm and mesoendoderm at 12 hpf. The expression pattern of *wnt11* overlaps with that of *wnt4a* in the floorplate at 16 and 24 hpf and with that of *wnt11f2* in the developing somites at 24 hpf. Of note, *wnt11* transcripts are also expressed in the heart tube at 24 hpf.^24^ In studying hair cell orientation, Navajas Acedo et al^45^ reported a parallel role of PCP and Wnt pathway genes in regulating hair cell orientations in zebrafish neuromasts. Mutation in *wnt11* disrupts hair cell orientation in neuromasts. However, heterozygous *wnt11* and *wnt11f2^tz216^* double mutants do not exhibit defects in hair cells, indicating that these two paralogs do not interact.

## Concluding Remarks and Perspectives

The *wnt11f2* mutant zebrafish is an important animal model that can help in understanding bone formation and the role of Wnt signaling pathways in this process.

Obviously, wnt11f2 is extremely important during gastrulation by enabling correct convergence–extension cell movements by activating the noncanonical Dvl/RhoA pathway.^24^ The *wnt11f2* mutant gastrulation phenotype is completely penetrant, whereas the eye phenotype is much more variable. This is also true for the deformities observed in the cranial cartilage, which were associated with the eye phenotype.

Future research should focus on the direct and indirect partners and signaling pathways that lead to the eye phenotype in homozygous mutants.

The *wnt11f2* is involved in the canonical and noncanonical Wnt signaling pathway, which is associated with cyclopia, and this defect is also observed in case of disruption of Shh and Nodal pathways in zebrafish.^46–48^ Probably there is an interaction between these different signaling pathways in zebrafish, but further investigations are needed.

In humans, cyclopia is a rare form of holoprosencephaly, and different forms of holoprosencephaly affect ∼1 in 15,000 human live births^49^ and as many as 1 in 250 human fetus.^50^

Holoprosencephaly is commonly due to Shh pathway dysfunction, but the Nodal pathway is also involved.^51^ Even if Wnt11f2 is absent in human, the noncanonical Dvl/RhoA pathway could be interesting to study to better understand holoprosencephaly. Moreover, *wnt11f2* remains an important tool to study Wnt signaling in early development.

In this study, we show that the heterozygous *wnt11f2* mutants, although normal in their general development, skeletal morphogenesis, and reproductive capacity, present a significantly more highly mineralized bone skeleton.

Several rare genetic disorders with skeletal effects, like osteopetrosis and sclerosing bone dysplasia, are associated with generalized bone mineral density increase. Many of the HBM diseases are monogenic disorders related to the Wnt signaling pathway.^52^ With this, we believe that the *wnt11f2* zebrafish is a great model to better understand HBM etiology and to screen new bone anabolic drugs.

Interestingly, we have recently shown that the heterozygous loss-of-function mutation of *WNT11* in humans exhibits the opposite phenotype.^53^ In a model of a *WNT11* heterozygous mutant human osteoblast-like cell line generated by CRISPR-Cas9, we showed that the noncanonical pathway acts upstream of the canonical pathway, since in the absence of *WNT11*, the canonical pathway could not be rescued^53^ as was also observed in *wnt11f2^tx226^* mutant zebrafish.^5^ This finding combined with our finding of HBM in *wnt11f2* heterozygous zebrafish suggests that the noncanonical Wnt pathway is key to bone differentiation independent of bone patterning. Therefore, the phenotypic characterization of *wnt11f2^tx226^* heterozygous adult would provide some insights into the potential genetic and cellular mechanisms of *wnt11f2^tx226^* function in bone.

In conclusion, we show that the noncanonical Wnt pathway acts as an inhibitory factor in bone mineralization in adults, and propose the heterozygous *wnt11f2* mutant as a model to better understand HBM phenotype.

## Supplementary Material

Supplemental data
