# Supplementary material for: wnt11f2 Zebrafish, an Animal Model for Development and New Insights in Bone Formation
Source: Zebrafish. 2023 Feb 14;20(1):1–9. doi: 10.1089/zeb.2022.0042 (PMC9968865; doi:10.1089/zeb.2022.0042)
Supplement: Supplemental data [file Supp_Data.docx]

**Supplementary data**

In this review we added 3 novel figures from our lab.

**Material and Methods.**

**Lineages**

*wnt11^tz216^* and *wnt11^tx226^* heterozygous were acquired from EZRC crossed with AB wild type. Wild types, heterozygous and homozygous used in this publication were obtained after a heterozygous crossing.

**Genotyping**

All the animals used in the experiments came from a het x het crossing and were genotyped after each experiment. For alcian blue experiment whole larvae was used. For RT-qPCR and microCT, DNA was extracted from the tail. Larvae or dissection were placed in 0.2 ml Eppendorf tubes, containing 100 µl of 20 mM NaOH. These samples were incubated at 95°C for 20 min, cooled down to 4°C, and neutralized using 20 µl of 1 M Tris-HCl for DNA extraction. Primers were design to flank the mutations. For the *wnt11^tz216^* zebrafish lineage, 5’-AGGCAGCGTTTGTGTTTTCT-3’and 5’-TCCACAGAAGACTGGCATCA-3’ primers were used for PCR amplification and BtsCI enzyme was used for genotype since the enzyme cuts the wild type. Primers for *wnt11^tx226^* 5’-AAATGTCATGGCGTTTCTGG-3’and 5’-CTCACCGGATTACTGCACA-3’ were used for PCR and genotyping was done using HPLC.

**Alcian blue staining**

To better understand the role *wnt11f2* in zebrafish cartilage formation, we performed alcian blue staining. 10 dpf in *wnt11f2^tx226^* larvae were fixed in 4% PFA for 2h at room temperature and rinsed several times with PBST. Cartilage was stained overnight in 10 mM MgCl_2_, 80% EtOH and 0.04% Alcian blue. The larvae were washed in different concentrations of ethanol (80%, 50%, 25%) to remove excess staining. Pigmentation was bleached in a H_2_O_2_ solution (H_2_O_2_ 3%, KOH 0.5%) and finally the larvae were rinsed 3 times in a solution of 25% glycerol / 0.1% KOH and 50% glycerol, 0.1% KOH and finally stored in this solution at 4°C (1). Pictures of the stained larvae (n=30) were taking using a Nikon Eclipse 50i microscope. Head measurements were made using ImageJ software.

**Gene expression analysis**

Total RNA was extracted from the head of 6 dpf *wnt11f2^tx226^* wild type, heterozygous and homozygous in pool of 3 already genotyped larvae. RNA was extracted by using the GeneJET RNA purification kit (ThermoFisher, Waltham, MA, USA) according to the manufacturer's protocols. The absorbance of RNA was determined at a wavelength 260 and 280 nm with a NanoDrop-2000 system (ThermoFisher, Waltham, MA, USA). cDNA was synthesized from RNA by using high-capacity cDNA reverse transcription kit (Applied Biosystems, Foster City, CA, USA) and was used immediately or stored at -20°C. The primers used for RT-PCR are in Table 1. The PCR amplification system included the following: 2X SYBR Green PCR master mix (Applied Biosystems, Foster City, CA, USA), 0.4 µM forward and reverse primers, 50 ng cDNA, and sterile water for a total volume of 25 µl. The thermocycling conditions were 95°C for 10 min, 95°C denaturation for 15 sec, 60°C anneal/extend for 1 min, for 40 cycles. Dissociation curve analysis was performed at the end of the PCR cycles. Gene expression levels were normalized to GAPDH level in each sample and were determined by the 2−∆∆Cq method (2).

**Table 1**

Primers used for zebrafish RT-qPCR.

| Gene | Forward 5'-3' | Reverse 3'-5' | Ref |
| --- | --- | --- | --- |
| eef1a1l1 (end) | CTTCTCAGGCTGACTGTGC | CCGCTAGCATTACCCTCC | (3) |
| sp7 | GGATACGCCGCTGGGTCTA | TCCTGACAATTCGGGCAATC | (4) |
| bglap | ACTGCACCTGGAGACCTGAC | TTTATAGGCGGTGATGATTC | (5) |
| fgf8a | GCCGTAGACTAATCCGGACC | TTGTTGGCCAGAACTTGCAC | (4) |
| wnt11 | TCCTCACATTCCTGCTCCTGTC | TCTTCATCTTCATTGGGGCATC | (6) |
| wnt11f2 | CACAACAATGCTGTTGGCAGACAGGTG | GGAGATGGTGCTGATGTCTTGAAGACC | (7) |
| axin2 | ACCCTCGGACACTTCAAGGAA | TCACTGGCCCTTTTGAAGAAGTAT | (8) |
| smo | TGAAGACTCAGAAACTCAAGA | GACCAACGGAGCCTCGCATT | (9) |
| gpc4 | CAGCTCAAACCCTTCGGAGAC | CGCTACAGTACGGGCAGTATAACAT | (10) |
| gli1 | TTCTTGGTTTACTTGAAGGCAGAG | GCTCATTATTGATGTGATGCACC | (11) |

**Micro-computed tomography (MicroCT)**

Skeletal morphology and bone structures were assessed using a micro-CT. 16 Adult fish, at 10 months post-fertilization were fixed in 4% PFA for 24h and placed in a moist chamber during scanning. Zebrafish had their whole-body scans with a spatial resolution of 7 µm were acquired at 70 kV and 100 µA with a 0.5-mm aluminum filter at a Skyscan 1272 microCT (Bruker, Kontich, Belgium). Image reconstruction was done using CTVox and CTAn softwares from Bruker as described before (12).

**Statistical analysis**

The results are expressed as mean ± SEM. Statistical analysis involved using GraphPad Prism 9.0 for Windows (GraphPad Software, San Diego, CA, USA, www.graphpad.com). Two-way ANOVA or Student t test was used for statistical analysis. P< 0.05 was considered statistically significant.

**References**

1. Aceto J, Nourizadeh-Lillabadi R, Marée R, et al. Zebrafish Bone and General Physiology Are Differently Affected by Hormones or Changes in Gravity. PLoS One. 2015;10(6):e0126928.

2. Livak KJ, Schmittgen TD. Analysis of relative gene expression data using real-time quantitative PCR and the 2(-Delta Delta C(T)) Method. Methods. 2001;25(4):402-8.

3. McCurley AT, Callard GV. Characterization of housekeeping genes in zebrafish: male-female differences and effects of tissue type, developmental stage and chemical treatment. BMC Mol Biol. 2008;9:102.

4. Gebuijs IGE, Raterman ST, Metz JR, et al. Fgf8a mutation affects craniofacial development and skeletal gene expression in zebrafish larvae. Biology open. 2019;8(9).

5. Yang DC, Tsai CC, Liao YF, et al. Twist controls skeletal development and dorsoventral patterning by regulating runx2 in zebrafish. PLoS One. 2011;6(11):e27324.

6. Jing L, Lefebvre JL, Gordon LR, et al. Wnt signals organize synaptic prepattern and axon guidance through the zebrafish unplugged/MuSK receptor. Neuron. 2009;61(5):721-33.

7. Seo J, Asaoka Y, Nagai Y, et al. Negative regulation of wnt11 expression by Jnk signaling during zebrafish gastrulation. J Cell Biochem. 2010;110(4):1022-37.

8. Safian D, Bogerd J, Schulz RW. Igf3 activates β-catenin signaling to stimulate spermatogonial differentiation in zebrafish. J Endocrinol. 2018;238(3):245-57.

9. Tehrani Z, Lin S. Antagonistic interactions of hedgehog, Bmp and retinoic acid signals control zebrafish endocrine pancreas development. Development. 2011;138(4):631-40.

10. Hu B, Gao Y, Davies L, et al. Glypican 4 and Mmp14 interact in regulating the migration of anterior endodermal cells by limiting extracellular matrix deposition. Development. 2018;145(17).

11. Han Y, Xiong Y, Shi X, et al. Regulation of Gli ciliary localization and Hedgehog signaling by the PY-NLS/karyopherin-β2 nuclear import system. PLoS Biol. 2017;15(8):e2002063.

12. Fiedler IAK, Schmidt FN, Wölfel EM, et al. Severely Impaired Bone Material Quality in Chihuahua Zebrafish Resembles Classical Dominant Human Osteogenesis Imperfecta. J Bone Miner Res. 2018;33(8):1489-99.
